# Membrane shrinkage and cortex remodelling are predicted to work in harmony to retract blebs

**DOI:** 10.1098/rsos.150184

**Published:** 2015-07-29

**Authors:** Thomas E. Woolley, Eamonn A. Gaffney, Alain Goriely

**Affiliations:** University of Oxford, Andrew Wiles Building, Radcliffe Observatory Quarter, Woodstock Road, Oxford OX2 6GG, UK

**Keywords:** cell motility, blebbing, shell model, hysteresis

## Abstract

Numerous cell types undergo an oscillatory form of dynamics known as blebbing, whereby pressure-driven spherical protrusions of membrane (known as blebs) expand and contract over the cell's surface. Depending on the cell line, blebs play important roles in many different phenomena including mitosis and locomotion. The expansion phase of cellular blebbing has been mathematically modelled in detail. However, the active processes occurring during the retraction phase are not so well characterized. It is thought that blebs retract because a cortex reforms inside, and adheres to, the bleb membrane. This cortex is retracted into the cell and the attached bleb membrane follows. Using a computational model of a cell's membrane, cortex and interconnecting adhesions, we demonstrate that cortex retraction alone cannot account for bleb retraction and suggest that the mechanism works in tandem with membrane shrinking. Further, an emergent hysteresis loop is observed in the intracellular pressure, which suggests a potential mechanism through which a secondary bleb can be initiated as a primary bleb contracts.

## 1. Introduction

Many animal cells have the ability to produce large, dynamic protrusions such as lamellae, filopods, microspikes and pseudopods [1]. Each of these four protrusion types rely on the polymerization of actin filaments in order to push the cell membrane outwards. Often, cells use these membrane extensions to undergo motility [2]; however, they can also be involved in a number of other different phenomena, such as mitosis [3].

Here, we are interested in a specific protrusion type known as a cellular bleb, which plays an important role in the locomotion of tumour cells, embryonic cells and stem cells [4–7]. Unlike the previously mentioned ‘actin-driven protrusions’, blebs do not extend because of actin filaments pushing the membrane outwards. Instead, blebs occur when a cell's lipid membrane bilayer delaminates from its actin cortex. If a cell's internal pressure is higher than the external pressure, then the pressure difference induces a flow of the cell's cytosol driving the membrane away from the cell and into a spherical protrusion, known as a bleb [8]. This localized swelling requires additional membrane to cover the bleb, but it is not currently known how this extra membrane can be produced and removed as quickly as observed. It has been hypothesized that extra membrane stems from high levels of wrinkling in the cellular surface [9]. Alternatively, it has been suggested that endo- and exo-cytosis processes could account for the membrane activity through localized recruitment [10]. In both cases, the growth of the membrane can be modelled as an increase in reference configuration, which is the approach taken here.

**Figure 1.**
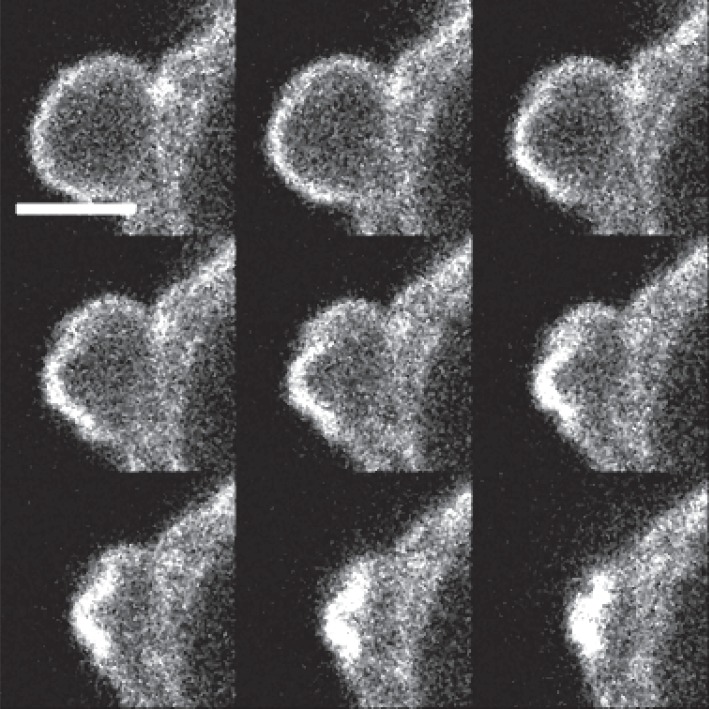
Confocal microscopy for a uniform timecourse showing a single bleb on a muscle stem cell being retracted over, approximately, 1 min. Used with permission from the Skeletal Muscle Development Group, University of Reading. The fluorescence highlights polymerized actin. Scale bar, 1.5 μm.

After approximately 10–30 s, the bleb expansion stops and an actin cortex reforms inside the bleb. Over a longer timescale of 1–2 min, the cell retracts the newly formed cortex within the bleb (which is coupled to the membrane), causing the bleb to shrink back into the cell, and allowing the process to begin again (figure 1). It is thought that myosin motors contract the cortex, causing it to shrink and, potentially, thicken [10]. It is this retraction phase that we are interested in modelling in this paper because bleb retraction is required for cells to move efficiently.

One should note that these ‘pressure-driven’ bleb protrusions, differ in mechanism, character and behaviour from actin-driven protrusions. For example, it has been shown that cellular motion dependent on blebs is much faster than many forms of lamellipodial movement. Further, blebbing cells are able to change the direction of their migration much quicker [11,12]. Owing to the fast-moving properties of the blebs over small spatial scales, it can be difficult to generate experimental insights into the physical processes that couple together to make blebbing possible, motivating the use of mathematical models to investigate the blebbing process.

Although blebbing is an extremely complex behaviour, the structural shape of a cell is thought to depend on three components: a flexible lipid bilayer membrane, a stiff actin cortex and adhesion proteins that couple these two structures [8]. Mathematical modelling offers a framework within which hypotheses can be tested, generating new predictions concerning the underlying mechanisms that control the blebbing expansion and retraction cycle. The hypothesis currently present in the biological literature is that blebs shrink simply due to the retraction of the bleb's reformed cortex, implying that the membrane is slave to the dynamics of the cortex [10]. Here, we test the biophysical and mechanical plausibility of experimentally suggested mechanisms that induce bleb retraction. However, an absence of molecular-level knowledge entails that we implement a phenomenological model of membrane and cortex retraction which captures the concept that myosin motors induce cortex shrinkage, but without detailed dynamics.

In terms of previous work, there are a number of different theoretical frameworks available considering diverse aspects of blebbing. Although some groups focus on using very high-level models to capture the entire blebbing expansion–retraction cycle [13,14], the majority deal with only the expansion phase of blebbing [15–19]. Our aim is to extend our previous model [20] to include mechanisms that will describe the retraction of spherical protrusions in order to investigate the expansion and retraction of blebbing in a unified mechanical model.

In our framework, the cell's membrane is an extensible, axisymmetric, elastic shell. The adhesion proteins that link the cortical cytoskeleton to the plasma membrane are thought to be members of the highly conserved ezrin–radixin–moesin (ERM) family [21]. Based on the work of Liu *et al.* [22], we model the adhesion molecules as piecewise neo-Hookean springs, in that their retraction force is a nonlinear function of their extension, up until a critical length. Beyond this critical extension, the adhesion molecules detach from the membrane, leaving the cortex and membrane no longer connected. Finally, the cortex is represented simply as a stiff elastic structure in which the adhesions are fixed.

Other frameworks for the cell membrane do exist; for example, it can be treated as a highly viscous fluid. However, we encompass these features within the solid mechanics framework as viscosity can be represented by a membrane with an evolving reference configuration. Equally, the growth of the membrane through a change in the arc-length kinematically captures all possible internal effects such as growth by addition of new material, resorption and fluid-like properties.

We begin in §2 by reproducing the key equations of the previously presented shell model of a bleb [15,16,20] and extend it to include the production of a new cortex in the bleb, the retraction of this new cortex and membrane shrinking. The initial results in §3 demonstrate that cortex retraction cannot produce bleb retraction on its own. Cortex retraction is then coupled to membrane shrinkage and it is observed that we are able to reproduce the observed bleb retraction, as well as produce membrane wrinkling depending on the ratio of timescales between the cortex retraction and membrane shrinking mechanism. Finally, in §4, we summarize the results and suggest how bleb retraction may lead to the initiation of further blebs, thus allowing cells to undergo self-consistent cyclical bleb dynamics.

## 2. Mathematical framework

The geometry and shell mechanics [23] are defined as in previous articles [16] and the pertinent equation system coupling the membrane, adhesions and cortex is briefly recapitulated here and explained below. A brief overview of the variables can be found in table 1 and further detail can be found in appendix A. The equations are

2.1 $$\begin{array}{rl} & \mathrm{geometry}\left\{ \begin{array}{l} \frac{\partial y}{\partial \sigma }=\lambda_{s}\cos(\theta ),\\ \frac{\partial z}{\partial \sigma }=-\lambda_{s}\sin(\theta ),\\ \frac{\partial \theta }{\partial \sigma }=\lambda_{s}\kappa_{s},\\ \frac{\partial s}{\partial \sigma }=\lambda_{s}, \end{array}\right. \end{array}$$

2.2 $$\begin{array}{rl} & \mathrm{principal}\;\mathrm{curvatures}\left\{ \begin{array}{l} \frac{\partial \kappa_{s}}{\partial \sigma }=\frac{\lambda_{s}}{y}\left(\cos(\theta )(\kappa_{s}-\kappa_{\phi })-\frac{Q_{s}}{M}\right),\\ \kappa_{\phi }=\frac{\sin(\theta )}{y}, \end{array}\right. \end{array}$$

2.3 $$\begin{array}{rl} & \mathrm{tangential}\;\mathrm{force}\;\mathrm{balance}\;\frac{\partial T_{s}}{\partial \sigma }=\lambda_{s}(t_{\phi }\cos(\theta )+Q_{s}\kappa_{s}), \end{array}$$

2.4 $$\begin{array}{rl} & \mathrm{normal}\;\mathrm{force}\;\mathrm{balance}\;\frac{\partial Q_{s}}{\partial \sigma }=\lambda_{s}((\Delta P-FC)y-\kappa_{\phi }yt_{\phi }-\kappa_{s}T_{s}), \end{array}$$

2.5 $$\begin{array}{rl} \;\mathrm{and}\; & \mathrm{adhesion}\;\mathrm{force}\left\{ \begin{array}{l} F(\sigma )=\kappa \left(\alpha E^{2}(\sigma )-\frac{\beta }{E(\sigma )}\right),\\ E(\sigma )=\sqrt{{\left(z(\sigma )-z_{c}(\sigma )\right)}^{2}+{\left(y(\sigma )-y_{c}(\sigma )\right)}^{2}}. \end{array}\right. \end{array}$$

To close the equation, system-suitable boundary conditions and constitutive equations need to be specified (see appendix A and A.1).

**Table 1.** Reference table for the variables and parameters in system (2.1)–(2.5). See text for further details.

name	description
variables that alter as the simulations progress	
*y*	vertical coordinate of the solution profile
*z*	horizontal coordinate of the solution profile
$\bar{y\;}\;$	vertical coordinate of the reference configuration
$\bar{z}$	horizontal coordinate of the reference configuration
*y*_c_	vertical coordinate of the cortex
*z*_c_	horizontal coordinate of the cortex
*θ*	solution profile normal angle measured anticlockwise from the *z*-axis
*s*	solution profile arc length
*σ*	reference configuration arc length
*κ*_s_	longitudinal principal curvature
*κ*_*ϕ*_	azimuthal principal curvature
λ_s_	arc length stretch ratio
*T*_s_=*yt*_s_	scaled form of the surface tension, *t*_s_, along the arc length
*t*_*ϕ*_	surface tension along the azimuthal coordinate
*Q*_s_=*yq*_s_	scaled form of the normal shear stress, *q*_s_
parameters that are constant throughout a simulation	
Δ*P*	pressure difference across the membrane
*C*	concentration of adhesions
*F*	adhesion force
*E*	adhesion extension
*E*_0_	adhesion resting length
*E*_c_	adhesion breaking length
*κ*	neo-Hookean spring constant
*α* and *β*	intrinsic adhesion properties scaling the force–extension relationship
*η*_1_	rate of cortex retraction
*η*_2_	rate of membrane retraction
*ρ*	radius of the initial reference configuration sphere
*σ*_0_	initial reference configuration arc length
*μ*	relative extensibility of the membrane in the azimuthal and longitudinal directions

**Figure 2.**
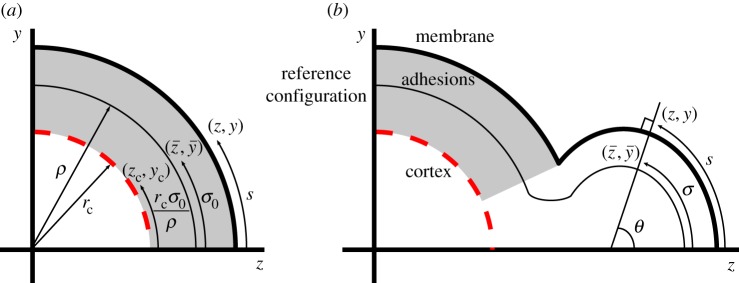
Definition of geometric variables concerning the coupling of the membrane, (*z*,*y*), the reference configuration, $\left(\bar{z},\bar{y\;}\;\right)$, and the cortex, (*z*_c_,*y*_c_). (*a*) The initial set-up where the membrane is adhered uniformly to a porous cortex. (*b*) The profile of the system once adhesions near the *z*-axis have been ablated and the reference configuration is allowed to grow. *θ* is the outward pointing normal angle between the membrane and the *z*-axis. See text for further details.

Equation set (2.1) defines the axisymmetrical geometry of the shell around the axis of rotational symmetry, here taken to be the *z*-axis (figure 2). As the two-dimensional shell is axisymmetric, we only need to consider a one-dimensional cross-section, at which point the radial coordinates can be related to the standard rectilinear Cartesian coordinates. Specifically, a reference configuration, $\left(\bar{z},\bar{y\;}\;\right)$, corresponding to the unstressed state, is parametrized by its arc length *σ* and measured from the intercept of the curve with the *z*-axis.

Initially, the reference configuration is a sphere of radius *ρ*, thus *σ*=*σ*_0_∈[0,*ρπ*]. The bleb production simulations are initiated by removing adhesions at the front of the cell, around *σ*=0. After the initial ablation of adhesions, the membrane deformation arises through reference configuration remodelling. This means that the arc length and profile of the reference configuration are able to update according to some postulated evolution rule. Critically, we only remodel the reference configuration within the unadhered region, $\sigma \in [0,\hat{\sigma }]$. Using the biologically demonstrated fact that strains are small [24], we fix the reference configuration update rule to be linear. Explicitly, if $\bar{y\;}\;(\sigma ,t)$ and *σ*=*σ*(*σ*_0_,*t*) are the profile of the reference configuration and corresponding arc length at time *t*, respectively, then

2.6 $$\begin{array}{rl} \frac{\partial \bar{y\;}\;}{\partial t}(\sigma ,t) & =\eta_{1}(y(\sigma ,t)-\bar{y\;}\;(\sigma ,t)), \end{array}$$

2.7 $$\begin{array}{rl} \bar{y\;}\;(\sigma ,0) & =\rho \sin\left(\frac{\sigma_{0}}{\rho }\right), \end{array}$$

and

2.8 $$\begin{array}{rl} \frac{\partial \sigma }{\partial t} & =\eta_{1}(s-\sigma ), \end{array}$$

2.9 $$\begin{array}{rl} \sigma (\sigma_{0},0) & =\sigma_{0}\in [0,{\hat{\sigma }}_{0}]. \end{array}$$

Once the equilibrium state of system (2.1)–(2.5) has been found, $\bar{y\;}\;$ and *σ* are updated. These new values for the reference configuration and arc length are then substituted back into the equations (2.1)–(2.5) and boundary conditions and the system is solved again. Critically, growth of the reference configuration ensures that the membrane does not stretch too far as it is well known that membrane tears after only a 4% area stretch [9] (see [15] for further details).

The solution configuration represents the shape that the reference configuration takes once it has been pressurized and is defined by the horizontal and vertical coordinates (*z*,*y*). Furthermore, *s* measures the arc length of the solution configuration and *θ* is the outward pointing normal angle of the membrane measured anticlockwise from the *z*-axis. Finally, in order to complete the geometric definition, we define *κ*_s_ and *κ*_*ϕ*_ through equation set (2.2), respectively, to be the principal curvatures of an axisymmetric surface.

Time evolution is applied through assuming that we are able to step the system through adiabatic approximations, while updating the reference configuration. Thus, during each iteration of the simulation, all forces acting on the shell are assumed to balance as summarized by equations (2.3) and (2.4), where the surface tensions *t*_s_ and *t*_*ϕ*_ are given in appendix A. The pressure difference, Δ*P*, is a Lagrange multiplier for the constraint of constant cell volume. Further, because the shell can support a small, but non-zero amount of bending, we define *M* in equation (2.2) to be the membrane bending modulus and note that there may be non-zero normal shear stresses, *q*_s_, which act along the membrane's normal direction. To aid efficient numerical simulation of the system, we formulate the model in terms of *T*_s_=*yt*_s_ and *Q*_s_=*yq*_s_, which allows us to remove singularities from all but the equation for *κ*_*ϕ*_.

**Figure 3.**
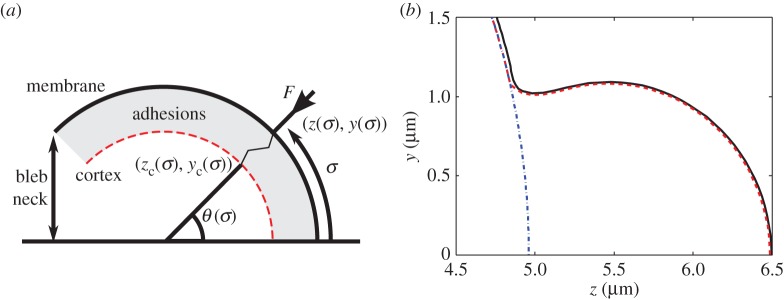
Creating a new cortex. (*a*) Schematic diagram illustrating the calculation of the reformed cortex location. (*b*) Blebbed cell showing the membrane as a solid (black) line. The old cortex, which was a sphere of radius *r*_c_= 4.98 μm, is the dashed and dotted (blue) line. The new cortex, calculated using equation (2.14), is the dashed (red) line, just beneath the membrane. The resting length is *E*_0_=0.01 μm.

Equation set (2.5) defines the relationship between the adhesion resistance force and the adhesion extension. Although the relationship between extension and force could be approximated by many different nonlinear equations, we have shown [20] that variations in the relationship have very little effect on the resulting behaviour of the system. This insensitivity to the force–extension relationship is because the extension range of the adhesions is very small. Thus, we adopt a constitutive law inspired from the uni-axial extension of a neo-Hookean material [25]. This choice captures the correct geometric behaviour in large deformations. In particular, it produces an infinite force when the adhesion's length tends to zero, which prevents the cortex and the membrane from penetrating each other. The parameters *α* and *β* are chosen such that the resting length is *E*_0_=10^−2^ μm and a Hookean constant close to this length is *κ*. Solving these conditions simultaneously,

2.10 $$F(10^{-2}\;\text{μ}m)=0\;\mathrm{and}\;\frac{\partial F}{\partial E}(10^{-2}\;\text{μ}m)=\kappa$$

provides us with the constants $\alpha =\frac{100}{3}\;\text{μ}m^{-1}$ and $\beta =\frac{1}{30000}\;\text{μ}m^{2}$, respectively. Note that the sign of the repulsive force is negative (without loss of generality) due to our arbitrary choice of force direction (see force vector of the adhesions in figure 3*a*). Also observe that since *F* is force per adhesion it is multiplied by an adhesion concentration, *C*, providing a force per area (pN μm^−2^), which counter-acts the pressure gradient. Finally, it should be noted that we are focused on the retraction phase of the blebbing cycle. For simulations focusing on how the membrane peels away from the cortex in the neck region, we direct the reader to our previous work [20].

In order to calculate the extension through equation (2.5), we need to know the position of the cortex. During bleb expansion, the cortex is assumed be a stiff sphere that contracts by a small amount, thus its coordinates are $\left(z_{c},y_{c})=(r_{c}\cos(\sigma_{0}/\rho ),r_{c}\sin(\sigma_{0}/\rho )\right)$, where *r*_c_ is the radius of the cortex and *ρ* is the radius of the initial spherical reference configuration (figure 2).

Cortex contraction is simulated by reducing the cortex radius by a constant, small amount, *δ*, after each iteration. The reason we choose to reduce the radius by a constant amount each time is because its reduction is extremely small (of the order of 10–20 nm, which is below the resolution of current confocal microscopy). Algebraically, this is

2.11 $$\frac{\partial r_{c}}{\partial t}=-\delta .$$

Alternatively, if the pressure from the cytosol acts on the actin skeleton as well as the membrane then, instead of the cortex contraction being an active process, cortex contraction could occur passively due to the bleb expansion reducing the pressure, which, in turn, would cause the actin skeleton to contract with *δ* ultimately expressible in terms of the cortex stiffness. In either case, because the radial change is so small we choose to approximate the cortex contraction through a linear decrease in radius.

We define the key parameters for the blebbing process illustrated in figure 2. Blebbing is initiated by removing adhesions within the region $\sigma_{0}\in [0,{\hat{\sigma }}_{0})$. During the simulation further adhesions may break. The point, along the arc length, at which the membrane transitions from adhered to the cortex to unadhered is denoted $\hat{\sigma }$. The neck width is $y(\hat{\sigma })$ and the bleb extension distance is $z(0)-z(\hat{\sigma })$. Finally, the widest point of the bleb satisfies ∂*y*(*σ*)/∂*z*=0, for $\sigma \in [0,\hat{\sigma })$ if such a point exists, otherwise the widest point is at $y(\hat{\sigma })$.

As demonstrated previously [20], equations (2.1)–(2.5) produce accurate bleb morphologies with the width of the bleb's neck smaller than the bleb's maximum width, known as small-necked blebs (figure 3*b*). The simulation of these blebs progresses as follows: adhesions are removed from the region $\left[0,{\hat{\sigma }}_{0}\right)$. This adhesion removal mimics experiments performed by Tinevez *et al.* [18], where adhesions were ablated using a laser causing blebs to appear. After this initial ablation of adhesion, the membrane deformation arises through reference configuration remodelling. For a given reference configuration, the solution configuration of system (2.1)–(2.5) is found. The reference configuration is updated using the new solution and the iteration process of finding a solution and updating the reference configuration is repeated.

### 2.1 Bleb retraction

Using the mathematical formulation presented in §2, we are able to iterate the system through adiabatic steps of membrane growth in order to produce a blebbed protrusion that matches the observed spherical-cap shape (figure 3*b*). We take this small-necked bleb as the initial condition for the retraction process. The retraction is initiated by creating a new cortex and adhering it to the membrane such that the adhesions are at their resting lengths (figure 3). Explicitly, because the adhesions align along the membrane normal direction, *θ*, the cortex coordinates, (*z*_c_(*σ*),*y*_c_(*σ*)), at a given point *σ* are the solutions to (figure 3*a*)

2.12 $${\left(y-y_{c}\right)}^{2}+{\left(z-z_{c}\right)}^{2}=E_{0}^{2}$$

and

2.13 $$\tan(\theta )(z_{c}-z)+y=y_{c},$$

namely

2.14 $$y_{c}=y-E_{0}|\sin(\theta )|\;\mathrm{and}\;z_{c}=z-\frac{E_{0}|\sin(\theta )|}{\tan(\theta )}.$$

Note that due to the high localization of the cortex reconfiguration, we only insert new cortex and adhesions in the blebbed region. The adhesions in the rest of the cell start from the initial expanded state and are allowed to relax during the simulation.

Once the new cortex has been formed adiabatic iterations are simulated, once again. However, whereas the bleb is formed by increasing the membrane's arc length, bleb retraction is produced through a reduction of the cortex and membrane arc lengths. Explicitly, the cortex is returned to its original configuration through the following equations:

2.15 $$\frac{\partial z_{c}}{\partial t}=\eta_{1}\left(r_{c}\cos\left(\frac{\sigma_{0}}{\rho }\right)-z_{c}\right)$$

and

2.16 $$\frac{\partial y_{c}}{\partial t}=\eta_{1}\left(r_{c}\sin\left(\frac{\sigma_{0}}{\rho }\right)-y_{c}\right).$$

Equally, the membrane is also updated through the following equations:

2.17 $$\frac{\partial \sigma }{\partial t}=\eta_{2}(\sigma_{0}-\sigma )$$

and

2.18 $$\frac{\partial \bar{y\;}\;}{\partial t}=\eta_{2}\left(\rho \sin\left(\frac{\sigma_{0}}{\rho }\right)-\bar{y\;}\;\right).$$

Observe that equations (2.15) and (2.16) will cause the cortex in the bleb to retract into the cell, while the cortex in the cell body expands slightly due to the fact that blebs are created through cortex contraction (see equation (2.11)). Simultaneously, equations (2.17) and (2.18) will cause the membrane to shrink back to its initial length.

The two additional mechanisms of cortex retraction and membrane shrinking are associated with timescales *η*_1_ and *η*_2_, respectively (see equations (2.15)–(2.18)), through which the speed of each mechanism can be controlled. We are able to consider each mechanism individually, through setting one of the timescales to zero, as well as their coupled interactions.

As the bleb is retracted, the system is effectively always in quasi-steady state as the system is overdamped. Hence retraction is driven by remodelling timescales. Equally, the impact of any heterogeneity in the thickness of the cortex is unclear, because we cannot be sure that it is accompanied by enhanced myosin aggregation and, thus, greater contractile forces, or if a thicker cortex would be harder to manipulate and, thus, slower to retract. Without further information, we have presented the simplest case of constant heterogeneous structures.

Furthermore, the modelled process of cortex retraction is phenomenological—the cortex within the modelling framework has a memory of its original configuration, approximating the observations that the cortex does retract along the given path [10]. Equally, although the membrane is known to shrink, it is also seen to wrinkle. In §3, we will be able to show that large wrinkles are seen if the cortex shrinks faster than the membrane (figure 7). The appearance of wrinkles in this case is to be expected as it has been shown that when a small substrate is attached to a larger membrane a buckling instability occurs in the membrane [26]. However, the solid mechanical framework we are using is unable to resolve the extremely small spatial creases that occur in the membrane as the bleb shrinks. In particular, we expect that the continuum approximation of the membrane is not valid at the small length scales of the highly oscillatory membrane wrinkles and we suggest that a discrete model of the lipid molecules would be needed to capture the wrinkling detail. Thus, understanding these limitations and without further data on how the cortex and membrane actually contract we use the current update rules as a way of encapsulating and approximating these unknown details without capturing molecular-level resolution.

Note that although membranes cannot support compressive stresses, there is a well-defined wrinkling theory for membranes that allows for stress computation (but not the shape) [27]. However, if we also take into account the contribution of the cytoskeleton, then we obtain a theory of shells that can support stresses and, in principle, wrinkles can be computed. However, there is insufficient experimental data, on both the effective bending modulus and the wrinkles (they are observed in some experiments but not controlled with respect to any accessible parameters), to warrant a detailed analysis of wrinkles.

## 3. Results

In all solutions, unless otherwise stated, the parameters are as given in table 2. The value of the ratio *η*_1_/*η*_2_ can be found in the caption of each simulation. The parameter values have been taken from a wide range of blebbing and membrane literature [12,22,24,28–30] and offer a scale of magnitude, if not an exact quantity.

**Table 2.** Parameter values used in all simulations, unless otherwise stated.

parameter	value
*α*	$\frac{100}{3}\;\text{μ}m^{-1}$
*β*	$\frac{1}{30000}\;\text{μ}m^{2}$
*A*	400 pN μm^−1^
*μ*	$\frac{1}{2}$
*M*	10^−2^ pN μm
*E*_0_	10^−2^ μm
*E*_c_	4×10^−2^ μm
*κC*	4×10^3^ pN μm^−3^
Δ*P*	20 pN μm^−2^ (initial value)

Before we discuss the results of bleb retraction, we first, briefly, address the matter of simulation work flow and, in particular, bleb production. The whole simulation can be envisioned as an iterated root finding problem. Specifically, upon initialization, system (2.1)–(2.5) is solved as a boundary value problem (boundary constraints are discussed in appendix A(A.1) for various different values of Δ*P*. The value of Δ*P* is iterated until the difference between the volume of the output solution and the initial volume constant is below a given tolerance. Once this tolerance is achieved the reference configuration and cortex are updated, if needed. Since each iteration only modifies the solution a small amount, we would expect the next solution state to be approximately similar to the previous solution state. Thus, the updated reference configuration and cortex curves are fed back into the root finding algorithm, along with the previous solution state. The algorithm uses the previous solution as an initialization state, around which to search for the new solution. Throughout the simulation, it was ensured that the numerical errors were never greater than the errors included due to the linearization of the boundary points (data not shown).

How the reference configuration and cortex curves are updated depends on whether the bleb is expanding or contracting. During the expansion phase, we demonstrated that small-necked blebs can be formed through two very different methods [20]: either the membrane is allowed to undergo localized growth, or the cell undergoes global cortex contraction. In either case, equations (2.6)–(2.9) are used to update the reference configuration of the membrane. Although there are multiple ways of producing a bleb, we propose only one method of retracting a bleb using equations (2.15)–(2.18), discussed in §2(a). The results in this paper are based on retracting blebs that have been produced through global cortex contraction. However, the retraction mechanism has been applied to the case of localized growth as well, where it also works and most results are not significantly different. The only result that is different between these two initialization methods is presented in the discussion of hysteresis in §3(e), where it is seen that initiation by global cortex contraction can offer emergent properties that are not seen in the case that blebs are initiated by localized membrane growth.

### 3.1 Cortex retraction only

As stated in §2(a), retraction of the blebs relies on the interacting timescales of cortex retraction and membrane shrinking. We begin this section by presenting the case in which membrane shrinkage does not occur, i.e. *η*_2_=0. Our aim is to theoretically demonstrate that cortex retraction is not solely capable of shrinking a bleb.

**Figure 4.**
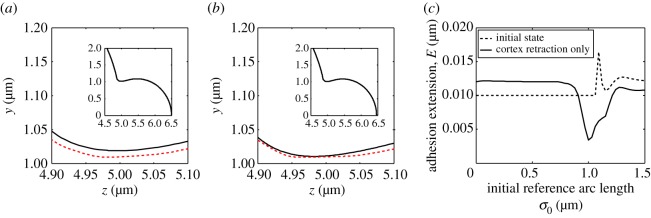
Result of retracting the cortex only. In (*a*,*b*) and all following cell profile images, the plotting scheme remains the same. The solid (black) line represents the membrane, while the dashed (red) line represents the cortex. (*a*) The initial state of the bleb with regenerated cortex, before it begins to retract. (*b*) Membrane and cortex profiles during cortex retraction. Note that as the simulation progresses the membrane begins to squeeze the cortex neck. The insets in (*a*,*b*) show the full cell profile and illustrate that the bleb hardly retracts before problems arise in the neck. (*c*) Extension of the adhesions for the profiles illustrated in images (*a*,*b*). The dashed lines illustrate that, as intended, the cortex in the blebbed region in (*a*) is initialized beneath the membrane such that the new adhesions in the bleb are at their natural resting length. The solid line shows that the adhesions in (*b*) are compressed in the neck region, whereas they are being stretched at the front of the membrane. In addition to the parameters fixed in the text, the timescale of membrane shrinking is *η*_2_=0 s^−1^, while *η*_1_>0 s^−1^ and, thus, $\eta_{1}/\eta_{2}=\infty$.

Figure 4 demonstrates the problems that occur when only cortex retraction occurs. As the simulation progresses, the cortex begins to pull the bleb towards the cell, with the adhesions becoming stretched at the front of the cell, near *σ*=0. However, owing to having a bleb neck smaller than the widest part of the bleb, the membrane in the bleb neck region begins to move towards the cortex, thereby compressing the adhesions in this region. Since the adhesions have a natural resting length of *E*_0_=0.01 μm, they are able to undergo some compression. However, as shown in figure 4*c* the adhesions in the neck region are being crushed between the membrane and cortex. This is of course unphysical, because, as noted in §2, the repelling force provided by the adhesions becomes very large as the adhesions are compressed. This increased force in the neck region would cause the membrane to crush the cortex, which is physically not possible.

### 3.2 Membrane shrinking only

**Figure 5.**
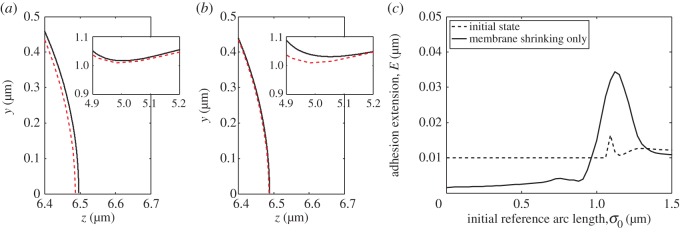
Result of shrinking the membrane only. (*a*) The initial state of the bleb's membrane and cortex at the bleb front and neck (shown in the inset). Compare with figure 4*a*. (*b*) Membrane and cortex profiles during membrane shrinking. Note that as the simulation progresses the membrane begins to squeeze the cortex at the front of the bleb, whereas the membrane is being pulled away from the cortex in the neck of the bleb (shown in the inset). (*c*) Extension of the adhesion for the profiles illustrated in images (*a*,*b*). The solid line characterizes the profiles seen in (*b*). Namely, the adhesions are heavily squeezed in the bleb front, whereas they are stretched in the neck region. In addition to the parameters fixed in §3, *η*_1_/*η*_2_=0.

Blebs also do not contract if cortex retraction is stopped and only membrane shrinking occurs. This should come as no surprise, primarily because the cortex forms a barrier, beyond which the membrane cannot move. However, by comparing the adhesion extension results from the cortex retraction simulation (figure 4*c*) with the adhesion extension results from membrane shrinking simulations (figure 5*c*) we observe that these two mechanisms have opposite effects on the evolution of the adhesion curves. Namely, in the case that only the cortex retraction is allowed, adhesions at the front are stretched while the adhesions in the neck are compressed. Antithetically, in the case that only the membrane shrinking is allowed, the adhesions at the front of the bleb are compressed and the adhesions in the neck are stretched. Thus by combining cortex retraction and membrane shrinking appropriately, there is the possibility that the bleb might retract without producing a situation where any of the adhesions are stretched, or compressed, beyond realistic expectations.

### 3.3 Coupling cortex retraction with membrane shrinking

**Figure 6.**
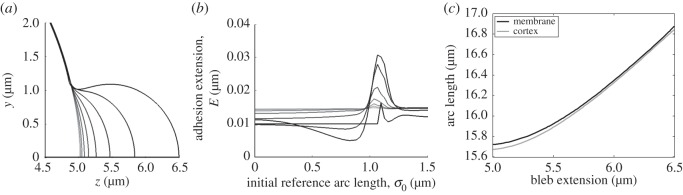
Coupling membrane shrinking and cortex retraction together. (*a*) Cell profile of the retracting bleb, the membrane lines becomes lighter to show the passage of time. The displayed time points are uniformly spaced over the simulation. (*b*) Adhesion extension curves corresponding to the profiles in (*a*). (*c*) The arc lengths of the cortex and membrane closely match each other as they are retracted. In addition to the parameters fixed in the text, $\eta_{1}/\eta_{2}=\frac{1}{2}$.

In figure 6, both cortex retraction and membrane shrinking are active and we clearly see that the bleb can be retracted. Moreover, we see that although the adhesions are not homogeneous in length during the simulation they are neither stretched more than the critical length, *E*_c_, nor are they compressed more than their resting length, *E*_0_, anywhere in the bleb. Note that as the bleb is finally retracted into the cell the adhesion lengths do homogenize as it approaches the spherical membrane solution, from which the bleb was first initiated.

Although we have shown that bleb retraction is possible using cortex remodelling and membrane shrinking, figures 4*c* and 5*c* suggest that this is only mechanically consistent with adhesion dynamics for a certain range of values of *η*_1_/*η*_2_. If *η*_1_/*η*_2_≪1, then the bleb retraction process is dominated by membrane shrinking, leading to the case shown in §3(b), where the adhesions at the front of the bleb are heavily compressed. Similarly, if *η*_1_/*η*_2_≫1, then the simulation is dominated by cortex retraction leading to compressed adhesions in the neck.

### 3.4 Wrinkling

**Figure 7.**
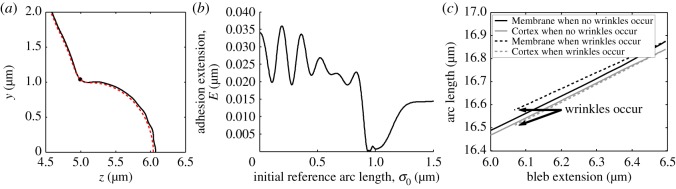
If cortex retraction is faster than membrane shrinkage, wrinkles in the bleb's membrane can appear. (*a*) Cell profile. (*b*) Adhesion extension illustrating the oscillatory nature of the membrane on the bleb. (*c*) Comparing the arc lengths of the cortex and membrane of the wrinkled solution (dashed line) with those of the retracted simulation (solid line) from figure 6. We see that the membrane and cortex arc lengths start to diverge during the wrinkling simulation (dashed lines), whereas the retracted simulation arc lengths track each other (solid lines). The additional parameter in the wrinkled simulation is *η*_1_/*η*_2_=2.

In the case that *η*_1_/*η*_2_ is around unity then the bleb can be contracted completely; however, by increasing the ratio further wrinkling can occur, as seen in figure 7*a*,*b*. As discussed in §2(a), these wrinkles appear because the arc length of the membrane is not shrinking as fast as the cortex's arc length. This concept is firstly evidenced in figure 7*c*, where we observe that in the case of bleb retraction (with $\eta_{1}/\eta_{2}=\frac{1}{2}$) the curves representing the arc lengths of the membrane and cortex match each other closely (solid line of figures 6*c* and 7*c*). Secondly, when *η*_1_/*η*_2_=2 wrinkles occur and the arc lengths of the membrane and cortex diverge (dashed line of figure 7*c*). Since the membrane is larger than the cortex to which it is attached, the unwrinkled state becomes unstable and thus membrane oscillations appear.

### 3.5 Expansion and retraction

**Figure 8.**
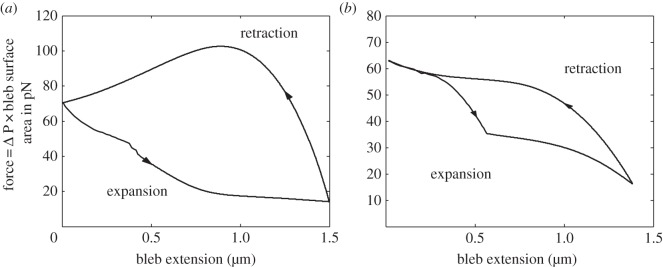
Force–extension curves for the expansion and retraction of blebs from different initiation mechanisms. (*a*) Bleb created through global cortex contraction. (*b*) Bleb created through localized membrane growth. In both cases, as a bleb expands, the cellular pressure (and, hence, force acting on the bleb) drops because of the assumption of constant solution volume. Conversely, as the bleb retracts the pressure increases. Note that hysteresis is present in the force–extension blebbing cycle. Parameters are the same as in figure 6.

Our final result links both the expanding and retraction phases. In figure 8, we consider the force–extension curve produced when blebs are initiated in two different ways. Figure 8*a* illustrates the evolution of the force that is present when the bleb is produced through global cortex retraction. This method has been used throughout the paper and is described in §2. Alternatively, figure 8*b* illustrates the force–extension curve in the case that the bleb is initiated through a local membrane growth mechanism. Although not explicitly discussed here in detail, extended examination of bleb production through local membrane growth can be found in our previous work [20]. The essential difference between the two bleb production mechanisms is that in the case of local membrane growth, the cortex does not undergo global contraction, instead it remains stationary. Furthermore, the membrane only grows within a fixed region, even if the membrane and cortex peel further apart. This can be compared with the global cortex contraction method, where the membrane is assumed to grow wherever the membrane and cortex are separated.

As we have discussed in previous articles [15,16,20], the intracellular pressure is one of the best measures of cellular dynamics. Figure 8 demonstrates that certain features of the force–extension curve are independent of the initialization mechanism. Namely, bleb expansion greatly reduces the intracellular pressure and, thus, the expansion force acting on the bleb, which matches the predictions of our previous work [15,16,20]. Further, we observe that as the bleb is retracted the cell repressurizes to its initial pressure difference, confirming the cyclical nature of the blebbing dynamics. Note also that the expansion–retraction oscillation forms a hysteresis loop. This is particularly pertinent in figure 8*a* since, for a given expansion, the force acting on a contracting bleb is always larger than the one acting on an expanding bleb.

Critically, figure 8 also presents a clear difference between the two initiation mechanisms. Note that in figure 8*a* the force increase during the retraction phase is not monotonic. This non-monotonicity in the force occurs due to the bleb being created through cortex contraction. As the bleb retracts, the entire cell relaxes back to its original spherical state, namely, the bleb shrinks and the cell body expands. Since the cell remodelling speed is assumed to be proportional to the difference between the reference configuration and the initial spherical state (see equations (2.15)–(2.18)), the bleb retraction will be quicker than the cell body expansion, because the bleb is further removed from the spherical state. Thus, as the bleb is retracted the reference configuration volume will evolve to be slightly smaller than its initial value, causing the pressure to increase above the initial pressure difference. However, as the cell body finally relaxes to the initial spherical state the pressure decreases to the original pressure difference.

This non-monotonicity in force increase does not occur when blebs are retracted in the local growth case (figure 8*b*), because the reference state volume is never smaller than its initial value. However, what is seen is that as the bleb expands initially adhesion breaking occurs, causing the membrane to peel away from the cortex. This extra unadhered membrane deviates away from the cortex and causes the pressure difference, and therefore the force, to fall quickly. Once the bleb has extended to approximately 0.5 μm, the pressure has dropped to a sufficient level such that no more adhesions break. Thus, the pressure drop after the extension of 0.5 μm arises purely from the bleb growing due to localized membrane growth. Overall, figure 8 allows us to demonstrate the ability of force, and in particular intracellular pressure, to act as an excellent proxy measure for the dynamics of the cell.

Note that the area enclosed inside the hysteresis loop of figure 8*a* is calculated to be 77.9 pN μm=77.9×10^−18^ J. This energy represents an estimate for the energy dissipated during a single bleb cycle. Further, taking 1 min to be a characteristic timescale of one cycle allows us to suggest that the power output of a single bleb is 1.3×10^−18^ W. These values are consistent with the adhesion energies estimated by Charras *et al.* [10].

## 4. Summary and conclusion

Extensive mathematical modelling work has been performed on the expansion phase of cell protrusions known as blebs. Here, we have developed a mathematical model for the retraction of these spherical, pressure-driven expansions. The framework couples an axisymmetric, elastic shell to a stiff reconfiguring surface through nonlinear springs that can break and reform. These three components model the membrane, the actin skeleton and interconnecting ezrin adhesions, respectively. Blebs are initiated by locally removing adhesions around the axis of axisymmetry. High intracellular pressure causes a bleb to extend from the ablated region, which is devoid of actin and adhesions. Once the bleb has extended to approximately 1.5 μm, bleb growth is stopped within the model and the actin cortex and adhesions are treated as having reformed within the bleb, which, save for a small delay, is a representation of the observations presented in figure 1. From this state, we investigated which mechanisms are able to successfully retract the bleb. Specifically, we demonstrated that membrane shrinking and cortex retraction must be carefully coupled.

If only one mechanism is considered, either cortex retraction or membrane shrinkage, we find that local regions of highly compressed adhesions are created, while adhesions in other regions are stretched. Critically, the adhesion regions that are compressed and stretched through the cortex retraction mechanism are exactly the regions that are stretched and compressed through the membrane shrinking mechanism. Thus, these two mechanisms can balance each other, ensuring that no region is stretched or compressed beyond physical constraints. Fortunately, this is consistent with observation that if cortex retraction is inhibited then blebs are unable to retract [31]. Furthermore, our model predicts that if membrane shrinking was separately inhibited bleb retraction would not occur, since either retraction stops or the membrane ruptures (which is implicitly assumed to induce cell death and stop bleb retraction).

Although these results may appear intuitively obvious and partially known through experimentation, the fact that our mathematical framework is able to reproduce them is extremely positive and represents an *a posteriori* evaluation that we are modelling (at least some of) the dominant mechanisms that are important in the blebbing process. Further, the model has allowed us to investigate and explain how these mechanisms breakdown in the case that blebs do not retract. Moreover, we have been able to predict that successful bleb retraction only occurs within a relatively small region of values for the ratio *η*_1_/*η*_2_, which is a measure of cortex retraction timescale to membrane shrinking timescale. This fine tuning in the coupling suggests that the mechanisms maybe jointly regulated. Equally, the model has allowed us to find, and explain the presence of, hysteresis loops in the blebbing force profiles, an idea which, to the authors' knowledge, has not previously been suggested.

The demonstration of hysteresis in the expansion and retraction loop of figure 8*a* allows us to offer an explanation behind the high frequency of blebs seen in numerous cell lines. Since for a given bleb extension distance the force acting on the cell is higher during the retraction phase than the expansion phase, this suggests that a new bleb could occur before the last one has fully retracted. Further, the force–extension curve shown in figure 8*a* offers a mechanism which initiates the next bleb formation. As the bleb retracts, the pressure difference, and therefore the force, increases beyond the initial spherical cell resting value. This increase in force coupled with stochastic adhesion binding and unbinding may trigger a local delamination and, thus, lead to the formation of a new bleb somewhere on the cell. This suggests that blebs can stimulate further blebbing events in a continual cascade of expansions and retractions. This result highlights that cortex contraction and expansion are important for predictions of such dynamics.

Through this and previous work [15,16,20], we have produced a complete mathematical framework of bleb expansion and retraction. Our solid mechanics model does have its limitation; for example, the geometry is specified to be axisymmetric, thus we are only able to consider blebs along the central axis of symmetry [16]. However, modelling single blebs is justified as our simple model has allowed us to understand the important factors pertaining to the expanding and contracting blebbing cycle, which in the future can be extended to further more complex bleb interactions.

Our aim throughout this body of work was to investigate factors constraining the size of the protrusions, which is highly correlated with their action. In particular, this paper focuses on investigating the ability of a cell to retract its blebs. Although our framework contains all the basic mechanics that allow the cell to bleb cyclically, there is still extensive work to be done in elucidating the mechanisms behind bleb initiation and cell polarization. Further, this model only supports axisymmetric protrusions and, thus, we will be looking to generalize our system to include multiple blebs over the entirety of the cell. Equally, this work depends on the assumption that the blebbing dynamics are in an adiabatic limit. The importance of this adiabatic assumption can be investigated through the addition of a hydrodynamic description for the cytosol, which in turn could be used to produce a temporally evolving blebbing system.

Even though the present framework is simple, it allows us to reach conclusions as to how blebbing is regulated. Firstly, the expansion phase of the blebbing process is dominated by membrane growth. This growth is essential in maintaining membrane stretches that are less than 4%. Secondly, although we have maintained a constant volume throughout all of this work, the pressure drop produced by a single bleb would seriously inhibit further bleb production, until the original bleb is significantly retracted. Experimentally, it is observed that multiple blebs can expand and contract simultaneously. We also note that the predictions of a drop in intracellular pressure with blebbing suggests the need for quantitative experimental measures of pressure using, for example, nanoscale Fabry–Perot resonators [32]. The resulting data would fundamentally inform our mechanical understanding of blebbing, as would a careful assessment of whether cell volume is conserved. Finally, we have shown that both biologically motivated mechanisms of membrane shrinking and cortex retraction are needed to accomplish bleb shrinking completely and that these mechanisms are able to generate a non-trivial elastic hysteresis curve, which in turn suggests a mechanism for bleb-induced blebbing.

## Appendix A. Mathematical system

In further detail to §2, boundary conditions and constitutive relations need to be specified in order to close the system (2.1)–(2.5). Parameters and variables are defined in table 1. First we provide constitutive relations that couple the surface tensions *t*_s_ and *t*_*ϕ*_ to the reference and solution configurations. Explicitly, these are

A 1 $$t_{s}=A(\lambda_{s}^{2}+\mu \lambda_{\phi }^{2}-(1+\mu ))$$

and

A 2 $$t_{\phi }=A(\mu \lambda_{s}^{2}+\lambda_{\phi }^{2}-(1+\mu )),$$

where reference and solution membrane configurations are related by two stretch ratios. The first stretch ratio, given by

A 3 $$\lambda_{\phi }=\frac{y(\sigma )}{\bar{y\;}\;(\sigma )},$$

is the *radial stretch ratio*, which measures the axisymmetric deformation. The second is λ_s_, which is defined in equation system (2.1) and represents the *arc length stretch ratio*, which, in turn, characterizes the local stretching of the body coordinates with respect to arc length.

Further, the parameter *A* characterizes the elastic properties of the membrane and *μ* measures the relative extensibility of the membrane in the azimuthal and longitudinal directions [23]. We have also assumed that the bending moments are isotropic and proportional to the mean surface curvature, i.e.

A 4 $$m_{\phi }=m_{s}=M(\kappa_{s}+\kappa_{\phi }-K_{0}),$$

where *K*_0_ is the mean curvature of the reference configuration and *M* is the bending modulus.

**A.1. Boundary conditions**

Since equations (2.1)–(2.5) contain a singularity at *σ*_0_=0 and *πρ*, the accompanying boundary conditions are not trivial. We expand *y*, *θ*, *s*, *t*_s_ and λ_s_ in powers of *ϵ* near *σ*_0_=0 and in powers of *πρ*−*ϵ* near *σ*_0_=*πρ*. Here, we specify only the boundary conditions near *σ*_0_=0, with the conditions at *σ*_0_=*πρ* being similar.

The expansions have the form

A 5 $$y=y_{0}+\epsilon y_{1}+\epsilon^{2}y_{2}+\cdots ,$$

where the other variables are given *mutatis mutandis*. Since we are imposing that the *z*-axis is the axis of spherical symmetry, the cell cuts the *z*-axis at both boundary points; thus, we immediately fix *y*_0_=*θ*_0_=*T*_*s*0_=*Q*_*s*0_=*Q*_*s*1_=*s*_0_=0. On expanding the equations near *σ*_0_=0 we find that

A 6 $$\begin{array}{rl} y_{1} & =\lambda_{1}, \end{array}$$

A 7 $$\begin{array}{rl} \theta_{1} & =\kappa_{s0}\lambda_{1}, \end{array}$$

A 8 $$\begin{array}{rl} s_{1} & =\lambda_{1} \end{array}$$

A 9 $$\begin{array}{rl} \;\mathrm{and}\;T_{s1} & =\lambda_{1}A(\lambda_{1}^{2}\left(\frac{1}{{\bar{y\;}\;}_{1}^{2}}+\mu \right)-(1+\mu )), \end{array}$$

where λ_1_ satisfies the following quartic

A 10 $$0=\kappa_{s0}A\left(\frac{1}{{\bar{y\;}\;}_{1}^{2}}+\mu \right)\lambda_{1}^{4}-\frac{1}{2}(\Delta P+2\kappa_{s0}A(1+\mu )-F(0)C(0))\lambda_{1}^{2}+Q_{s2}^{2}.$$

By construction, we take the root that connects continuously to the real, positive root that exists when *Q*_*s*2_=0. Equations (A6)–(A9) form the four boundary conditions needed to be satisfied at *σ*_0_=*ϵ*≪1. The final three come from deriving analogous equations at *σ*_0_=*πρ*−*ϵ* and noting that we do not need to specify two boundary conditions for the variable *s*. This is because the equation for ∂*s*/∂*σ* decouples and can be determined as an initial value problem, once the rest of the system has been solved as a boundary value problem.
